# F221. SELECTIVE ATTENTION BIAS FOR FEAR STIMULI AND HALLUCINATION IN PATIENTS WITH SCHIZOPHRENIA: A PRELIMINARY STUDY

**DOI:** 10.1093/schbul/sby017.752

**Published:** 2018-04-01

**Authors:** Jiyun Yum, Han-Suk Kim, Ho Jun Seo

**Affiliations:** The Catholic University of Korea

## Abstract

**Background:**

Several studies have shown the association between affective dysregulation and severity of psychotic symptoms in schizophrenic patients. Attentional biases, which operate automatically to favor the processing of emotionally negative information in early stages of information processing, are known to play a causal role in the etiology of anxiety and other negative affective states. This study was conducted to evaluate the association between selective attention bias for fear stimuli and psychotic symptoms in patients with schizophrenia.

**Methods:**

A total of 66 patients with schizophrenia were included in the study. Attentional biases were measured with the dot-probe task with facial expression of neutral and fear emotional. To measure the psychotic features of the participants, the Positive and Negative Symptom Scale (PANSS), Psychotic Symptom Rating Scale (PSYRATS), the Scale to Assess Unawareness of Mental Disorder (SUMD), and Clinical Global Impression–Severity scale (CGI-S) were used.

**Results:**

Attentional vigilance scores were calculated by subtracting the median RT in congruent trials (dot at the position of the fear face) from the median RT in incongruent trials (dot at the position of the neutral face) Attentional vigilance scores was moderately correlated with the hallucination subscale of PSYTATS (r=0.268, p=0.029) in the participants. No correlation was found between selective attention bias and the scores of PANSS, PSYTATS-delusion, SUMD, and CGI-S. When the participants were divided into biased and non-biased groups by the attentional vigilance scores of +40 msec, no significant difference was found in the clinical measures. However, a statistical trend was found in hallucination severities between the biased and non-biased groups (p=0.092).

**Discussion:**

As a pilot study, the results suggest that the emotional information processing might affect the subjective severity of psychotic features in schizophrenia. Further study would be needed to clarify this association.

